# Cavernous Hemangioma of the Anal Canal Mimicking a Neoplasm: A Rare Cause of Lower Gastrointestinal Bleeding Successfully Treated With Transanal Excision

**DOI:** 10.7759/cureus.84214

**Published:** 2025-05-16

**Authors:** Eduardo Merchan, Ivan M Solis, Jhon Tapia, Ana M Nuñez, Álvaro Morillo Cox, Tatiana Fernandez Trokhimtchouk

**Affiliations:** 1 Colorectal Surgery, Hospital de Especialidades Carlos Andrade Marin, Quito, ECU; 2 General Surgery, Universidad Internacional del Ecuador, Quito, ECU; 3 General Surgery, Universidad de las Américas, Quito, ECU; 4 Surgery, Hospital de Especialidades Carlos Andrade Marin, Quito, ECU

**Keywords:** anal canal, anorectal mass, cavernous hemangioma, differential diagnosis, mri, rectal bleeding, transanal excision, vascular malformation

## Abstract

Cavernous hemangiomas of the anal canal are exceptionally rare vascular malformations that may present with chronic rectal bleeding and are often misdiagnosed as hemorrhoids or neoplastic lesions. We report the case of a 75-year-old female patient with a one-year history of painless rectal bleeding and a soft polypoid lesion located 2 cm from the anal verge, within the anterior wall of the anal canal. Magnetic resonance imaging (MRI) revealed a hyperintense pseudonodular lesion consistent with a vascular malformation. Histopathological examination after biopsy confirmed the diagnosis of cavernous hemangioma. Definitive treatment was achieved through transanal full-thickness excision, with complete resolution of symptoms and no recurrence at the three-month follow-up. This case highlights the importance of including vascular lesions in the differential diagnosis of anorectal bleeding and illustrates the utility of MRI and organ-preserving surgery in the management of anal canal hemangiomas.

## Introduction

Cavernous hemangiomas of the lower gastrointestinal tract are rare benign vascular lesions arising from the submucosal venous plexus, representing a minority among vascular malformations in this region [1, 2]. The rectosigmoid colon is the most frequently affected site, and while these lesions are typically diagnosed in younger individuals, they can present across a broad age spectrum [3, 4]. Clinically, rectal hemangiomas most often manifest as painless, recurrent rectal bleeding, although more severe presentations such as anemia or massive hemorrhage have been described [5, 6].

The rarity of these lesions, combined with their nonspecific endoscopic appearance and tendency to mimic more common pathologies such as internal hemorrhoids or anorectal tumors, frequently results in diagnostic delays or misdiagnosis [1, 2]. In fact, multiple reports have documented inappropriate interventions, including hemorrhoidectomy or anti-inflammatory treatment, prior to accurate identification of the underlying vascular nature of the lesion [3, 7].

Endoscopy is a first-line diagnostic tool, and biopsy is discouraged due to the risk of bleeding [2, 4]. Imaging modalities such as magnetic resonance imaging (MRI), especially T2-weighted sequences, can provide supportive evidence by demonstrating hyperintense, well-demarcated lesions consistent with vascular malformations [3].

Definitive treatment is guided by lesion size, depth, and symptom severity. While surgical excision has traditionally been the mainstay of therapy, minimally invasive techniques such as endoscopic submucosal dissection (ESD) and endoscopic full-thickness resection (EFTR) have emerged as alternatives in selected cases [5]. However, surgery remains a critical option, particularly when diagnostic uncertainty exists or malignancy cannot be excluded.

Herein, we report the case of a 75-year-old female patient with a cavernous hemangioma of the anal canal, initially suspected to be a neoplastic lesion. The case underscores the diagnostic challenges presented by this uncommon entity, highlights the role of imaging, and poses transanal surgical excision as the mainstay for definitive diagnosis and treatment.

## Case presentation

We present the case of a 75-year-old female patient, with no significant past medical or surgical history, referred to our department of coloproctology for evaluation of an anal mass. The patient reported intermittent rectal bleeding for the past year, without pain, altered bowel habits, or systemic symptoms. On digital rectal examination, a soft, mobile, polypoid lesion was palpated on the anterior wall of the anal canal, approximately 2 cm from the anal verge.

A prior colonoscopy report provided by the patient described an exophytic, bleeding lesion in the anal canal. Following our departmental protocol, a rigid rectosigmoidoscopy was performed, revealing a pink, polypoid lesion approximately 1.5 cm in diameter, located in the anterior aspect of the anal canal. Given the lesion’s appearance, a neoplastic etiology was initially suspected, and a biopsy was performed. Post-procedural bleeding was controlled with direct pressure and silver nitrate application.

Baseline laboratory tests, including complete blood count, serum biochemistry, coagulation profile, and tumor markers, were all within normal limits. Table 1 summarizes the patient's most relevant laboratory values.

**Table 1:** Summary of the patient's baseline laboratory results All values were within normal limits at the time of initial evaluation, including complete blood count, serum biochemistry, coagulation parameters, and tumor markers.

Test	Result	Reference range
Hemoglobin (g/dL)	16	12.0–15.5 (female)
Hematocrit (%)	51.8	36–46
White blood cells (×10⁹/L)	6.57	4.0–10.0
Neutrophils (%)	53.4	40–70
Lymphocytes (%)	35,8	20–45
Sodium (mmol/L)	142	135–145
Potassium (mmol/L)	4.3	3.5–5.1
Chloride (mmol/L)	100	98–107
Urea (mg/dL)	18.9	7–20
Creatinine (mg/dL)	0.8	0.6–1.2
International normalized ratio (INR)	1.09	0.8–1.2
Prothrombin time (s)	12	10–13
Thrombin time (s)	19	14–21
Carcinoembryonic antigen (CEA) (ng/mL)	3.27	<5.0
Cancer antigen (CA) 19-9 (U/mL)	12.89	<37
CA-125 (U/mL)	14.91	<35

**Figure 1:**
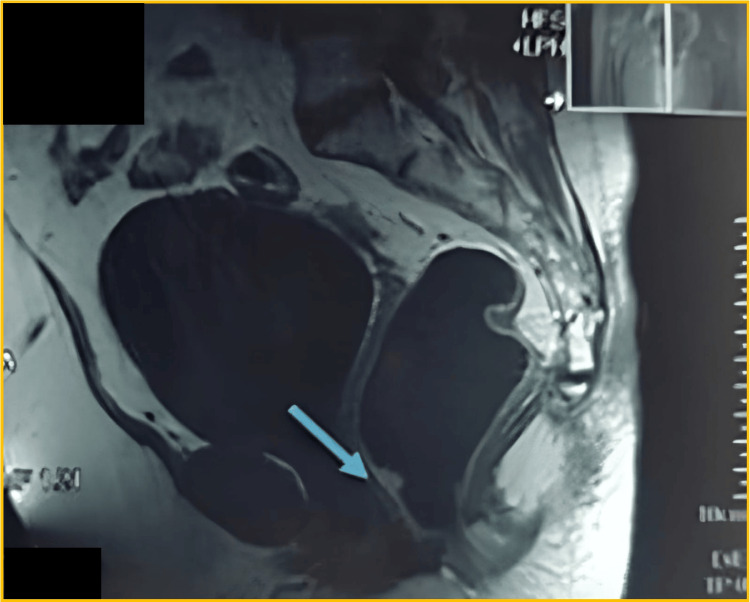
Pelvic MRI T2-weighted sagittal view of the pelvic MRI showing a hyperintense, pseudonodular lesion (blue arrow) arising from the anterior wall of the anal canal, approximately 2 cm from the anal verge. The lesion measures 1.5 cm in diameter and is consistent with a vascular malformation.

A pelvic MRI was ordered and demonstrated a pseudonodular lesion, 1.5 cm in size, arising from the anterior wall of the anal canal at 2 cm from the anal verge. The lesion appeared hyperintense on T2-weighted sequences, suggestive of a vascular nature (Figure 1). There were no additional abnormal findings.

**Figure 2:**
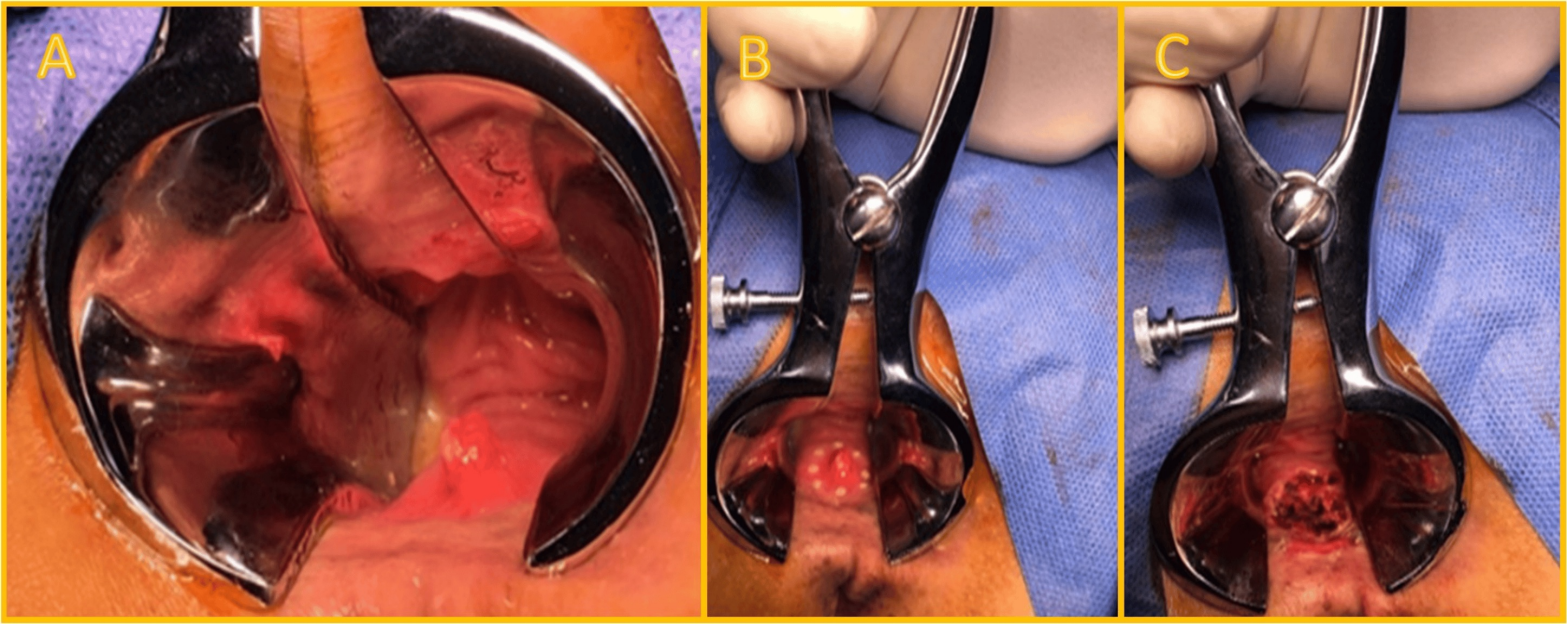
Intraoperative images of the transanal surgical resection (A) Visualization of the anterior wall of the anal canal using anoscopic exposure. The vascular lesion is clearly identifiable, appearing polypoid and hyperemic. (B) Circumferential marking of a 1 cm margin around the lesion prior to resection. (C) Post-resection view demonstrating the full-thickness excision site, with the surgical defect left open to heal by secondary intention.

Histopathological analysis of the biopsy confirmed the diagnosis of cavernous hemangioma. Given the potential for persistent bleeding and inability to completely rule out malignancy, the decision was made to proceed with surgical excision. A transanal full-thickness excision was performed under direct vision with anoscopic assistance, with the patient placed in the jackknife position. A 1 cm safety margin was marked circumferentially around the lesion, and excision was completed using an ultrasonic dissection device. The surgical defect was left open to heal by secondary intention, a common approach in the perianal region to allow adequate drainage and reduce the risk of hematoma or infection (Figure 2).

**Figure 3:**
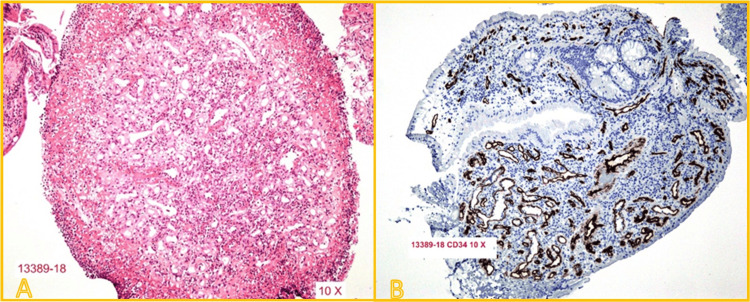
Histopathological examination of the resected lesion (A) Hematoxylin and eosin (H&E) staining (10×) reveals large, dilated, blood-filled vascular spaces lined by a single layer of flattened endothelial cells, consistent with cavernous hemangioma. (B) Immunohistochemistry for CD34 (10×) shows strong membranous positivity in the endothelial lining of the vascular spaces, confirming their vascular nature.

The postoperative course was uneventful. The patient was discharged on postoperative day 1. Final pathology confirmed complete resection of a cavernous hemangioma, with immunohistochemical staining positive for CD34 in vascular endothelium and a Ki-67 index of 2%, supporting a benign vascular lesion (Figure 3). Follow-up visits at three and 12 weeks included rigid rectosigmoidoscopy, which showed normal mucosal healing with no evidence of recurrence or residual lesion.

## Discussion

Cavernous hemangiomas of the anorectal region are exceedingly rare vascular lesions, accounting for less than 0.05% of all colorectal tumors, with most cases in the literature describing rectal involvement. Anal canal localization remains exceptionally uncommon. Moreover, low lesions may be missed during routine colonoscopy, which often bypasses the distal 2-3 cm, potentially delaying diagnosis [8,9]. These benign malformations originate from the submucosal venous plexus and are believed to result from congenital mesodermal sequestration during embryogenesis [2,9]. The most frequently affected site is the rectosigmoid junction, and clinical manifestations typically involve painless rectal bleeding, often intermittent, leading to chronic iron deficiency anemia or, less commonly, life-threatening hemorrhage [3,10].

In our patient, laboratory studies were within normal limits, including hemoglobin and coagulation profile. The absence of anemia, despite a one-year history of intermittent rectal bleeding, is noteworthy, as iron deficiency anemia is commonly described in vascular lesions of the gastrointestinal tract. While tumor markers were measured and found to be normal, they are not useful for the diagnosis or exclusion of anal canal hemangiomas.

The insidious onset and nonspecific nature of symptoms contribute to frequent diagnostic delays. Literature indicates that up to 80% of patients initially receive erroneous diagnoses, including hemorrhoids, inflammatory bowel disease, or neoplasms, leading to inappropriate therapies such as hemorrhoidectomy or steroid use [1,2,8]. Our case follows this pattern, with the lesion initially misinterpreted as a neoplasm and subjected to biopsy, which triggered bleeding, a known risk of attempting tissue diagnosis in vascular lesions [4,6].

These lesions may appear as bluish or violaceous submucosal masses, soft and compressible, sometimes with a polypoid component [7,10]. Biopsy is typically contraindicated due to the risk of hemorrhage, although in our case, histological diagnosis was fortuitously achieved from the initial biopsy without serious complications. The gold standard for preoperative characterization is MRI, which typically shows hyperintense signals on T2-weighted images with serpiginous or nodular enhancement, often pathognomonic for vascular malformations [3,6,9]. This imaging modality also assists in delineating sphincter involvement and planning for surgical or endoscopic resection.

While conservative measures such as embolization or sclerotherapy have been reported in the past, they are generally ineffective in the long term due to high recurrence rates and are therefore not commonly used in current practice [2,5]. Complete resection remains the mainstay of curative treatment. Historically, abdominoperineal resection (APR) was considered the definitive approach, particularly in diffuse or transmural disease involving the sphincter complex [3,6]. However, localized or pedunculated lesions, such as in our case, may be managed successfully with sphincter-preserving techniques, including transanal excision, EFTR, or, more recently, ESD [5,7].

The use of ESD in rectal hemangiomas remains limited to isolated case reports but represents a promising, less invasive alternative to surgery in well-selected patients [7]. Nonetheless, the choice between endoscopic and surgical management must account for lesion location, size, depth, vascularity, and diagnostic certainty. In our case, transanal full-thickness excision with ultrasonic dissection achieved curative resection with uneventful recovery and no evidence of recurrence at the three-month follow-up.

Histologically, cavernous hemangiomas consist of large, dilated vascular channels lined by flattened endothelium and separated by fibrous stroma. Immunohistochemistry is typically positive for endothelial markers such as CD34 and negative for proliferative markers, consistent with benign behavior [1,4,7].

This case underscores the importance of including vascular malformations in the differential diagnosis of rectal bleeding, particularly when lesions appear polypoid or atypical on endoscopy. Recognition of characteristic MRI features and avoidance of biopsy when vascular lesions are suspected are essential to reduce morbidity. Although rare, cavernous hemangioma of the anorectal canal should be considered when evaluating patients with unexplained rectal bleeding, especially in the absence of systemic or inflammatory findings.

## Conclusions

Cavernous hemangioma of the anal canal is a rare and often misdiagnosed cause of lower gastrointestinal bleeding. Its nonspecific presentation and frequent mimicry of more common anorectal pathologies, such as hemorrhoids or malignancy, pose significant diagnostic challenges. This case highlights the pivotal role of clinical suspicion, high-resolution imaging, and histopathological confirmation in achieving a correct diagnosis. MRI, particularly T2-weighted imaging, offers valuable non-invasive characterization of vascular lesions and should be considered in the preoperative workup of atypical anorectal masses.

While endoscopic techniques such as ESD may offer promising minimally invasive treatment options in selected cases, transanal surgical excision remains a safe and effective approach for localized lesions. Awareness of this uncommon entity is essential to avoid unnecessary interventions, prevent complications related to misdiagnosis, and guide appropriate, organ-preserving treatment.
